# Genetic factors associated with response to as-needed aflibercept therapy for typical neovascular age-related macular degeneration and polypoidal choroidal vasculopathy

**DOI:** 10.1038/s41598-020-64301-z

**Published:** 2020-04-28

**Authors:** Seigo Yoneyama, Yoichi Sakurada, Wataru Kikushima, Atsushi Sugiyama, Mio Matsubara, Yoshiko Fukuda, Naohiko Tanabe, Ravi Parikh, Fumihiko Mabuchi, Kenji Kashiwagi, Hiroyuki Iijima

**Affiliations:** 1 Department of Ophthalmology, University of Yamanashi, Chuo Yamanashi, Japan; 2 New York University School of Medicine, New York, NY USA; 3 Manhattan Retina and Eye Consultants, New York, NY USA

**Keywords:** Macular degeneration, Retinopathy of prematurity

## Abstract

In the present study, we investigated the association between susceptible genetic variants to age-related macular degeneration (AMD) and response to as-needed intravitreal aflibercept injection (IAI) therapy for exudative AMD including both typical neovascular AMD and polypoidal choroidal vasculopathy (PCV) over 12-months. A total of 234 patients with exudative AMD were initially treated with 3 monthly IAI and thereafter as-needed IAI over 12 months. Seven variants of 6 genes including *ARMS2* A69S (rs10490924), *CFH* (I62V:rs800292 and rs1329428), *C2-CFB-SKIV2L*(rs429608), *C3* (rs2241394), *CETP* (rs3764261) and *ADAMTS-9* (rs6795735) were genotyped for all participants using TaqMan technology. After adjusting for age, gender, baseline BCVA and AMD subtype, A (protective) allele of *C2-CFB-SKIV2L* rs429608 was associated with visual improvement at 12-month (P = 0.003). Retreatment was associated with T(risk) allele of *ARMS2* A69S (P = 2.0 × 10^−4^; hazard ratio: 2.18:95%CI: 1.47-3.24) and C(risk) allele of *CFH* rs1329428 (P = 2.0 × 10^−3^; hazard ratio: 1.74:95%CI: 1.16–2.59) after adjusting for the baseline confounders. The need for additional injections was also associated with T allele of *ARMS2* A69S (P = 1.0 × 10^−5^) and C allele of *CFH* rs1329428 (P = 3.0 × 10^−3^) after adjusting for the baseline confounders. The variants of *ARMS2* and *CFH are* informative for both physicians and patients to predict recurrence and to quantify the need for additional injections.

## Introduction

Advanced age-related macular degeneration (AMD), a leading cause of blindness in the industrialized countries of Asia^1^, is subdivided into choroidal neovascularization secondary to exudative AMD or geographic atrophy (GA). A recent clinic-based study reported that exudative AMD accounts for almost 95% of advanced AMD in the Japanese^2^. It has been reported that more than 20 genetic variants are associated with AMD through genome-wide association studies^3–5^. Of the genetic variants which make one more susceptible to AMD, variants in *ARMS2* and *CFH genes* have been reported to be strongly associated with AMD, followed by variants in *C2*-*CFB-SKIV2L* and *C3* genes^3^.

To date, intravitreal injections of anti-vascular endothelial growth factor (VEGF) agents have been first-line therapy for exudative AMD worldwide. VIEW1/2 study demonstrated that three monthly intravitreal aflibercept injection (IAI) followed by bimonthly IAI was effective for treating exudative AMD and was non-inferior to monthly intravitreal ranibizumab injection regarding best-corrected visual acuity (BCVA)^6^. *Pro re nata* (PRN) regimen is also referred to as-needed regimen, and several prospective studies have demonstrated that PRN regimen is almost equivalent as monthly dosing regimen in terms of visual improvement^7,8^. PRN may also be a more practical treatment strategy due to the burdens of treatment cost and frequent clinic visits.

In a recent genome-wide association study investigating the response to ranibizumab for exudative AMD among Japanese patients using a PRN regimen, no specific gene variants were associated with a significant improvement of visual acuity at 12 months. However, the *ARMS2* A69S variant was associated with need for retreatment^9^. To date, there have been no reports investigating differences in response to aflibercept for exudative AMD among genotypic variants.

In the present study, we investigated the genetic associations of response to as-needed IAI with visual outcomes and the need for additional injections after initial 3 monthly IAI and followed by as-needed IAI for exudative AMD during 12-month follow-up.

## Results

Table 1 shows baseline demographic and genetic characteristics of 234 patients composed of 118 patients with typical neovascular AMD and 116 patients with polypoidal choroidal vasculopathy (PCV). Compared with patients with PCV, patients with typical neovascular AMD were older (p = 3.5 × 10^−6^) and had higher T allele frequency of *ARMS2* A69S (p = 3.5 × 10^−3^). The fellow eye condition was no drusen (n = 96), pachydrusen (n = 20), soft drusen (n = 49), pseudodrusen(n = 32), and exudative AMD/scarring(n = 37).

**Table 1:** Demographic and genetic characteristics in patients with exudative ag-related macular degeneration.

	All patients (n = 234)	Typical neovascular AMD (n = 118)	PCV (n = 116)	P value
Age	74.9 ± 8.2	77.5 ± 8.0	72.2 ± 7.7	3.5 × 10^−6^
Male gender	170 (72.7%)	80 (67.8%)	90 (77.6%)	0.093
Need for retreatment	157 (67.1%)	81 (68.6%)	76 (65.5%)	0.31
Baseline BCVA (log MAR)	0.42 ± 0.37	0.51 ± 0.38	0.33 ± 0.33	4.0 × 10^−4^
***ARMS2*** **A69S (rs10490924)**				
TT	100(42.7%)	60(50.9%)	40(34.5%)	
TG	95(40.6%)	44(37.2%)	51(44.0%)	
GG	39(16.7%)	14(11.9%)	25(21.5%)	
T allele frequency	63.0%	69.5%	56.5%	3.5 × 10^−3^
***CFH*** **I62V (rs800292)**				
GG	134(57.3%)	71(60.2%)	63(54.3%)	
GA	88(37.6%)	42(35.6%)	46(39.7%)	
AA	12(5.1%)	5(4.2%)	7(6.0%)	
G allele frequency	76.1%	78.0%	74.1%	0.016
***CFH*** **(rs1329428)**				
CC	101(43.2%)	52(44.1%)	49(42.2%)	
CT	108(46.2%)	59(50.0%)	49(42.2%)	
TT	25(10.6%)	7(5.9%)	18(15.6%)	
C allele frequency	66.2%	69.1%	63.4%	0.19
***C2-CFB-SKIV2L*** **(rs429608)**				
AA	2(0.9%)	0	2(1.7%)	
AG	33(14.1%)	13 (11.0%)	20(17.2%)	
GG	199(85.0%)	105(89.0%)	94(81.1%)	
A allele frequency	7.9%	5.5%	10.3%	0.053
***C3*** **(rs2241394)**				
GG	1(0.4%)	0	1(0.9%)	
GC	27(11.5%)	14(11.9%)	13(11.2%)	
CC	206(88.0%)	104(88.1%)	102(87.9%)	
G allele frequency	6.2%	5.9%	6.5%	0.81
***CETP*** **(rs3764261)**				
TT	10(4.3%)	5(4.2%)	5(4.3%)	
TG	70(29.9%)	33(28.0%)	37(31.9%)	
GG	154(65.8%)	80(67.6%)	74(63.8%)	
T allele frequency	19.2%	18.2%	20.3%	0.58
***ADAMTS9*** **(rs6795735)**				
CC	4(1.7%)	1(0.8%)	3(2.6%)	
CT	55(23.5%)	29(24.6%)	26(22.4%)	
TT	175(74.8%)	88(74.6%)	87(75.0%)	
C allele frequency	13.5%	13.1%	13.8%	0.84

**Figure 1:**
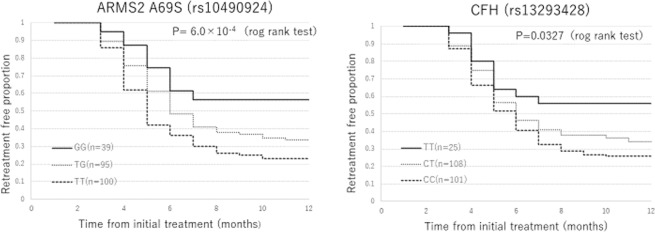
Retreatment free period from the initial injection according to *ARMS2* A69S and *CFH* rs1329428 genotypes. (**A**) Retreatment free period from the initial injection according to *ARMS2* A69S genotypes. Mean retreatment-free period after the initial injection was 9.0 ± 3.5 (95%CI:7.9–10.1), 7.6 ± 3.5 (95%CI:6.9–8.3), 6.5 ± 3.3 (95%CI:5.9–7.2) in GG genotype, TG genotype, TT genotype, respectively. There was a significant difference in retreatment-free period among *ARMS2* A69S genotypes (p = 6.0 × 10^−4^, log-rank test). (**B**) Retreatment free period from the initial injection according to *CFH* rs1329428 genotypes. Mean retreatment-free period after the initial injection was 8.8 ± 3.8 (95%CI:7.3–10.3), 7.5 ± 3.6 (95%CI:6.8–8.2), 6.9 ± 3.4 (95%CI:6.2–7.5) in TT genotype, CT genotype, CC genotype, respectively. There was a marginal significant difference in retreatment-free period among CFH rs1329428 genotypes (p = 0.0327, log-rank test).

Table 2 shows the demographic and genetic characteristics of patients with or without requiring retreatment. Of 234 patients, 157(67.1%) required the additional injection. Compared with patients without requiring the retreatment, patients requiring the retreatment were significantly older(p = 5.2 × 10^−4^) and had higher risk allele frequency of *ARMS2* A69S and *CFH* rs1329428(p = 2.0 × 10^−4^ and 2.0 × 10^−3^, respectively). Table 3 show number of additional intravitreal aflibercept injection according to genotypes in 7 variants of 6 genes. Number of additional injections was significantly different among *ARMS2* A69S and *CFH* rs1329428 (p = 1.2 × 10^−5^ and 4.0 × 10^−3^, respectively). Number of additional injections was significantly associated with risk allele of *ARMS2* A69S (risk allele: T allele) and *CFH* rs1329428 (risk allele: C allele) (p = 1.0 × 10^−5^ and 3.0 × 10^−3^, respectively). Figure 1 shows Kaplan-Meier estimator demonstrating retreatment-free period from the initial injection according to *ARMS2* A69S genotypes and *CFH* rs1329428 genotypes. A statistically significant difference was seen in retreatment-free period among *ARMS2* A69S genotypes (P = 6.0 × 10^−4^, log-rank test).

**Table 2:** Comparison of genetic characteristics between patients with or without retreatment.

	With retreatment (n = 157)	Without retreatment (n = 77)	Univariate p-value	Multivariate p-value	Odds ratio (95%CI)
Age	76.2 ± 7.8	72.1 ± 8.5	3.5 × 10^−6^	5.2 × 10^−4^(※)	1.06 (1.03–1.10)
Male gender	113(72.0%)	57(74.0%)	0.74	NA	NA
AMD subtype (neovascular AMD)	81(51.6%)	37(48.1%)	0.61	NA	NA
***ARMS2*** **A69S (rs10490924)**					
TT	77(49.0%)	23(29.9%)			
TG	63(40.1%)	32(41.6%)			
GG	17(10.9%)	22(28.5%)			
T allele frequency	69.1%	50.6%	1.0 × 10^−4^	2.0 × 10^−4^(※※)	2.18 (1.47–3.24)
***CFH*** **I62V (rs800292)**					
GG	96(61.1%)	38(49.4%)			
GA	55(35.0%)	33(42.9%)			
AA	6(3.8%)	6(7.3%)			
G allele frequency	78.7%	71.2%	0.060	NA	NA
***CFH*** **(rs1329428)**					
CC	75(47.8%)	26(33.7%)			
CT	71(45.2%)	37(48.1%)			
TT	11(7.0%)	14(18.2%)			
C allele frequency	71.3%	57.6%	6.8 × 10^−3^	2.0 × 10^−3^(※※)	1.74 (1.16–2.59)
***C2-CFB-SKIV2L*** **(rs429608)**					
AA	1(0.6%)	1(1.3%)			
AG	21(13.4%)	12(15.6%)			
GG	135(86.0%)	64(83.1%)			
A allele frequency	7.5%	7.6%	0.51	NA	NA
***C3*** **(rs2241394)**					
GG	0	1(1.3%)			
GC	16(10.2%)	11(14.3%)			
CC	141(89.8%)	65(84.4%)			
G allele frequency	4.5%	7.6%	0.16	NA	NA
***CETP*** **(rs3764261)**					
TT	8(5.1%)	2(2.6%)			
TG	46(29.3%)	24(31.2%)			
GG	103(65.6%)	51(66.2%)			
T allele frequency	18.7%	20.5%	0.69	NA	NA
***ADAMTS9*** **(rs6795735)**					
CC	0	4(5.2%)			
CT	39(24.8%)	16(20.8%)			
TT	118(75.2%)	57(74.0%)			
C allele frequency	10.8%	15.9%	0.35	NA	NA

(※)adjusting for gender, baseline BCVA, AMD subtype.

(※※) adjusting for age, gender, baseline BCVA, AMD subtype.

**Table 3:** Number of additional injections according to genotypes during 12-month.

		Number of additional treatments	P value	Adjusted p value
*ARMS2* A69S (rs10490924)	TT	2.66 ± 2.31		
	TG	1.84 ± 1.85		
	GG	1.05 ± 1.43	1.2 × 10^−5^	1.0 × 10^−5^(※)
*CFH* I62V (rs800292)	GG	2.31 ± 2.20		
	GA	1.78 ± 1.91		
	AA	1.33 ± 1.50	0.027	NA
*CFH* (rs1329428)	CC	2.45 ± 2.24		
	CT	1.89 ± 1.96		
	TT	1.24 ± 1.56	4.0 × 10^−3^	3.0 × 10^−3^(※)
*C2-CFB-SKIV2L* (rs429608)	AA	1.50 ± 2.12		
	AG	1.85 ± 1.95		
	GG	2.10 ± 2.10	0.46	NA
*C3* (rs2241394)	GG	0		
	GC	1.52 ± 1.93		
	CC	2.14 ± 2.09	0.085	NA
*CETP* (rs3764261)	TT	2.70 ± 1.57		
	TG	1.81 ± 1.88		
	GG	2.13 ± 2.19	0.81	NA
*ADAMTS9* (rs6795735)	CC	0		
	CT	2.58 ± 2.48		
	TT	1.94 ± 1.92	0.43	NA

(※) adjusting for age, gender, baseline BCVA, AMD subtype.

Table 4 shows baseline BCVA and BCVA improvement according to genotypes in 7 variants of 6 genes. Although there was not a significant difference in baseline BCVA among genotypes in the 7 variants, A allele in rs429608 of *C2-CFB-SKIV2L* was significantly associated with BCVA improvement at 12-month.

**Table 4:** Visual improvement according to genotypes at 12-month.

		Baseline BCVA	P value	BCVA improvement at 12month	P value	Adjusted p value
*ARMS2* A69S (rs10490924)	TT	0.42 ± 0.37		−0.12 ± 0.28		
	TG	0.49 ± 0.40		−0.20 ± 0.33		
	GG	0.30 ± 0.24	0.48	−0.14 ± 0.20	0.34	NA
*CFH* I62V (rs800292)	GG	0.41 ± 0.37		−0.15 ± 0.26		
	GA	0.43 ± 0.41		−0.16 ± 0.32		
	AA	0.54 ± 0.45	0.33	−0.25 ± 0.43	0.39	NA
*CFH* (rs1329428)	CC	0.42 ± 0.37		−0.17 ± 0.27		
	CT	0.43 ± 0.38		−0.15 ± 0.30		
	TT	0.40 ± 0.45	0.93	−0.14 ± 0.33	0.48	NA
*C2-CFB-SKIV2L* (rs429608)	AA	0.70 ± 0.42		−0.50 ± 0.14		
	AG	0.49 ± 0.44		−0.30 ± 0.37		
	GG	0.41 ± 0.36	0.12	−0.13 ± 0.27	1.0 × 10^−3^	3.0 × 10^−3^(※)
*C3* (rs2241394)	GG	0.7		−0.18		
	GC	0.52 ± 0.39		−0.18 ± 0.29		
	CC	0.41 ± 0.37	0.11	−0.15 ± 0.29	0.70	NA
*CETP* (rs3764261)	TT	0.27 ± 0.09		−0.04 ± 0.40		
	TG	0.39 ± 0.34		−0.18 ± 0.23		
	GG	0.45 ± 0.39	0.081	−0.16 ± 0.30	0.42	NA
*ADAMTS9* (rs6795735)	CC	0.33 ± 0.30		−0.14 ± 0.28		
	CT	0.35 ± 0.28		−0.09 ± 0.26		
	TT	0.45 ± 0.39	0.083	−0.18 ± 0.30	0.057	NA

(※)adjusting for age, gender, baseline BCVA, and AMD subtype.

## Discussion

To date, anti-VEGF therapy is a first-line treatment for exudative AMD worldwide; however, for each eye there may be a wide range of clinical responses as well as number of injections needed. In some patients, BCVA greatly improved without additional injections, while in other patients, BCVA deteriorated in spite of monthly injection over 12-months. Given that several genetic factors are associated with clinical phenotype in exudative AMD^10–13^, it would be reasonable to consider that differences in response to the treatment might contribute to genetic factors. Our study has found some genetic basis in the variable response as well as treatment burden among Japanese patients with macular neovascularization from typical neovascular AMD and PCV. We found that the A (protective) allele of *SKIV2L* rs429608 was associated with visual improvement at 12-months. Further, we found that the need for retreatment was associated with T(risk) allele of *ARMS2* A69S and the C(risk) allele of *CFH* rs1329428 after adjusting for the baseline confounders.

There have been several reports investigating the association between genetic variants and response to ranibizumab or bevacizumab for exudative AMD. In CATT study, none of the investigated variants (*CFH*, *ARMS2/HTRA1*, *C3*) were associated with clinical outcomes including visual outcomes, OCT parameters, number of additional injections^14^. A recent Japanese prospective study using ranibizumab PRN regimen demonstrated that risk allele of *ARMS2* A69S was associated with retreatment^9^. Several retrospective studies using ranibizumab also reported that risk allele of *ARMS2* was associated with retreatment or number of additional injections^15,16^. In the present study using aflibercept, our results were consistent with previous reports, but our study is the first to investigate the effect of aflibercept response among the aforementioned genetic variants. In addition to *ARMS2* A69S, risk (C) allele of *CFH* rs1329428 was associated with retreatment and number of additional injections. C allele of *CFH* rs1329428 is a susceptible variant to AMD; on the other hand, it has been reported that T allele of *CFH* rs1329428 is known as a genetic susceptible variant to central serous chorioretinopathy (CSC)^17–19^ and with choroidal vascular hyperpermeability (CVH) and subfoveal choroidal thickness in eyes with PCV^20^. G allele of *CFH* I62V is a responsible variants in the *CFH* region for exudative AMD in the Japanese population; however, interestingly, response to aflibercept therapy in terms of retreatment was associated with C allele of *CFH* rs1329428 rather than G allele of *CFH* I62V.

Pharmacogenetic genetic association was seen in terms of retreatment and number of additional IAI. Therefore, genotyping variants of *ARMS2* A69S and *CFH* rs1329428 would be informative for both patients and physicians to predict additional injections and number of additional injections. This would be important for patient education, informing follow up, and bring AMD care towards a more personalized approach to optimize visual outcomes.

Regarding the association between visual improvement and genetic variants, several studies demonstrated that none of variants were associated with visual improvement^9,14^ and other studies have shown the positive association between *ARMS2/HTRA* variants and visual improvement^21,22^. In the present study, there was a statistically significant association between *C2-CFB-SKIV2L* rs429608 genotypes and visual improvement. There was a wide range in baseline BCVA among rs429608 genotypes although it did not reach a statistically significance. Further studies would be needed to confirm or refute the present results.

Limitations of the current study include its retrospective nature of analysis and sample size and lack of information regarding symptoms prior to the initial presentation although the current sample is larger than prior retrospective genotype-clinical outcome studies in AMD. In the present study, we genotyped major risk allele in 7 variants of 6 genes; however, we cannot exclude the possibility that other susceptible variants to AMD are associated with aflibercept treatment response. A large-scale prospective genome-wide association study using aflibercept would be needed to confirm our tentative conclusion.

In summary, the A allele of *C2-CFB-SKIV2L* rs429608 was associated with visual improvement while the T allele of *ARMS2* A69S and C allele of *CFH* rs1329428 were associated with the need for retreatment and a greater number of additional injections during the first 12 months of PRN aflibercept therapy among treatment naïve exudative AMD of Japanese descent.

## Methods

### Subjects

We retrospectively reviewed the medical charts of 234 eyes (from 234 patients) with treatment naïve exudative AMD receiving 3 monthly intravitreal aflibercept (0.05 ml/2 mg) injections at the University of Yamanashi Hospital between 2013 January and 2018 September and completed 12-months of follow-up. We included the patients with exudative AMD including PCV and typical neovascular AMD. Eyes with typical neovascular AMD show type 1 or type 2 NV on SD-OCT and absence of polypoidal lesion on ICGA. This study was approved by the institutional review board at the University of Yamanashi and adhered to the tenets of Declaration of Helsinki. Written informed consent was obtained from each patient before treatment.

### Treatment and follow-up

Prior to the treatment, all patients had undergone comprehensive ophthalmic examinations including best-corrected visual acuity (BCVA) measurement using Landolt C chart, intraocular pressure measurement, slit-lamp biomicroscopy with 78 diopter contact lens, fundus color photography, spectral-domain optical coherence tomography(OCT) using Spectralis version 5.4 HRA + OCT (Heidelberg Engineering, Dossenheim, Germany) and fluorescein angiography(FA) and indocyanine green angiography(ICGA) using a confocal laser scanning system (HRA-2;Heidelberg Engineering, Dossenheim, Germany).The fellow eye condition was classified into 5 groups;1) no drusen,2) pachydrusen,3)soft drusen,4)pseudodrusen with or without soft drusen,5)exudative AMD/scarring based on the criteria as we previously described^23^.

All patients received 3-monthly intravitreal aflibercept injection (IAI) followed by monthly follow-up. BCVA and IOP measurement, biomicroscopy with or without 78D lens and a crosshair OCT scan were examined for all study eyes at each visit. If intraretinal/subretinal fluid was detected on SD-OCT or new macular hemorrhage were found on ophthalmoscopy, single additional intravitreal aflibercept injection was administrated.

### Genotyping

A peripheral blood sample (5 ml) was collected when baseline FA/ICGA was performed. Genomic DNA was purified using PureGene Isolation Kit (Gentra Systems, Minneapolis, US). The variants of *ARMS2* A69S(rs10490924), *CFH* I62V(rs800292), *CFH* rs1329428, *C2-CFB-SKIV2L* rs429608, *C3* rs2241394, *CETP* rs3764261, *ADAMTS9* rs6795735 were selected to genotype in the present study because these variants were reported to have a strong association with AMD in the Japanese population. Because the T allele of *CFH* rs1329428 was reported to be associated with central serous chorioretinopathy and choroidal vascular hyperpermeability in eyes with PCV^17,20^, the *CFH* I62V variant was also included in our genotyping analysis. Genotyping was performed using TaqMan genotyping assays with 7300/7500 Real-Time PCR Systems (Applied Biosystems, Foster City, CA) with manufacturers’ recommendation as we previously described^20,24^.

### Statistical analysis

Statistical analysis was performed using DR. SPSS for Windows (IBM, Tokyo, Japan). BCVA measured with decimal format using Landolt chart was converted to the logarithm of minimum angle of resolution (log MAR) for statistical analysis. Differences in categorical variables were examined using chi-square test. Difference in continuous variables between 2 groups and 3 groups were examined using Mann-Whitney U test and analysis of variance, respectively. Adjusted p-value was obtained using multivariate linear regression analysis. P-value less than 0.05 was considered statistically significant. In addition, we genotyped 7 genetic variants and assumed that these 7 genetic variants were statistically independent, we used the Bonferroni correction, which require less than 0.0071(0.05/7) to reach a statistical significance.
